# Cell cycle-related genes associate with sensitivity to hydrogen peroxide-induced toxicity

**DOI:** 10.1016/j.redox.2022.102234

**Published:** 2022-01-17

**Authors:** Sander Bekeschus, Grit Liebelt, Jonas Menz, Debora Singer, Kristian Wende, Anke Schmidt

**Affiliations:** a ZIK plasmatis, Leibniz Institute for Plasma Science and Technology (INP), Felix-Hausdorff-Str. 2, 17489, Greifswald, Germany; b Department of General, Visceral, Vascular, and Thorax Surgery, Greifswald University Medical Center, Felix-Hausdorff-Str. 2, 17475, Greifswald, Germany

**Keywords:** Cancer, Hydrogen peroxide, Oxidative stress, Reactive oxygen species, ROS

## Abstract

Reactive oxygen species (ROS) such as hydrogen peroxide (H_2_O_2_) are well-described agents in physiology and pathology. Chronic inflammation causes incessant H_2_O_2_ generation associated with disease occurrences such as diabetes, autoimmunity, and cancer. In cancer, conditioning of the tumor microenvironment, e.g., hypoxia and ROS generation, has been associated with disease outcomes and therapeutic efficacy. Many reports have investigated the roles of the action of H_2_O_2_ across many cell lines and disease models. The genes predisposing tumor cell lines to H_2_O_2_-mediated demise are less deciphered, however. To this end, we performed in-house transcriptional profiling of 35 cell lines and simultaneously investigated each cell line's H_2_O_2_ inhibitory concentration (IC_25_) based on metabolic activity. More than 100-fold differences were observed between the most resistant and sensitive cell lines. Correlation and gene ontology pathway analysis identified a rigid association with genes intertwined in cell cycle progression and proliferation, as such functional categories dominated the top ten significant processes. The ten most substantially correlating genes (Spearman r > 0.70 or < -0.70) were validated using qPCR, showing complete congruency with microarray analysis findings. Western blotting confirmed the correlation of cell cycle-related proteins negatively correlating with H_2_O_2_ IC_25_. Top genes related to ROS production or antioxidant defense were only modest in correlation (Spearman r > 0.40 or < -0.40). In conclusion, our in-house transcriptomic correlation analysis revealed a set of cell cycle-associated genes associated with *a priori* resistance or sensitivity to H_2_O_2_-induced cellular demise with the detailed and causative roles of individual genes remaining unclear.

## 1 Introduction

Reactive oxygen species (ROS) such as hydrogen peroxide (H_2_O_2_) are well-described agents in physiology and pathology. ROS are inevitable in life, and most organisms generate significant amounts of unintentionally or intentionally generated ROS, e.g., NADPH-oxidase (NOX)-produced superoxide and nitric oxide synthase (NOS)-produced nitric oxide, throughout their existence. At physiological concentration, such ROS participate in cellular signaling to respond to moderate extracellular and intracellular perturbations. At supraphysiological concentrations, irreversible modifications of biomolecules contribute to cellular dysfunction and demise. These two modes have been coined oxidative eustress and oxidative distress, respectively [1]. Notably, intentional ROS generation is employed by cells of the adaptive immune system for antimicrobial defense [2].

Being a long-lived non-radical ROS, H_2_O_2_ plays a central role in governing cellular processes. H_2_O_2_ is mainly generated via spontaneous or superoxide dismutase (SOD)-mediated conversion of superoxide. Chronic inflammation and H_2_O_2_ generation have been linked to both oncogenesis and tumor progression [3]. For instance, NOX-expressing cancers were shown to mimic features of wound healing and, by that driving angiogenesis and growth [4]. At the same time, detrimental levels of H_2_O_2_ have also been implicated in tumor control. Redox-active chemotherapeutic compounds were given a role in tumor therapy [5], and several approaches dwell on the idea that tumor cells have decreased capabilities of withstanding higher ROS levels [6], especially H_2_O_2_. Among such therapeutic approaches are, for instance, H_2_O_2_-guided chemodynamic therapy [7], H_2_O_2_-responsive photothermal and photodynamic therapy [8] and nanoparticles [9], secondary H_2_O_2_ generated via radiotherapy [10], and other physical modalities and technologies [11]. In general, radiotherapy, photodynamic therapy, and medical gas plasma technology have been noted to deliver or locally generate tumor-toxic concentrations of several types of ROS that aid in tumor control [[12], [13], [14]].

The importance of ROS, including H_2_O_2,_ is undisputed in oncology. However, it is less clear which transcriptional profiles are associated with either sensitivity or resistance to H_2_O_2_-mediated cytotoxicity. This study used 35 cell lines from various organs and performed in-house transcriptomics and H_2_O_2_ inhibitory concentration (IC)_25_ assessments from the same cultures. A large share of target genes identified to correlate highly (Spearman r > 0.70 or < -0.70) with H_2_O_2_-induced cytotoxicity are involved in cell cycle progression and control, and the most relevant genes were validated using qPCR and western blotting, underlining their involvement in translating H_2_O_2_-mediated tumor growth control.

## 2 Results

**Fig. 1:**
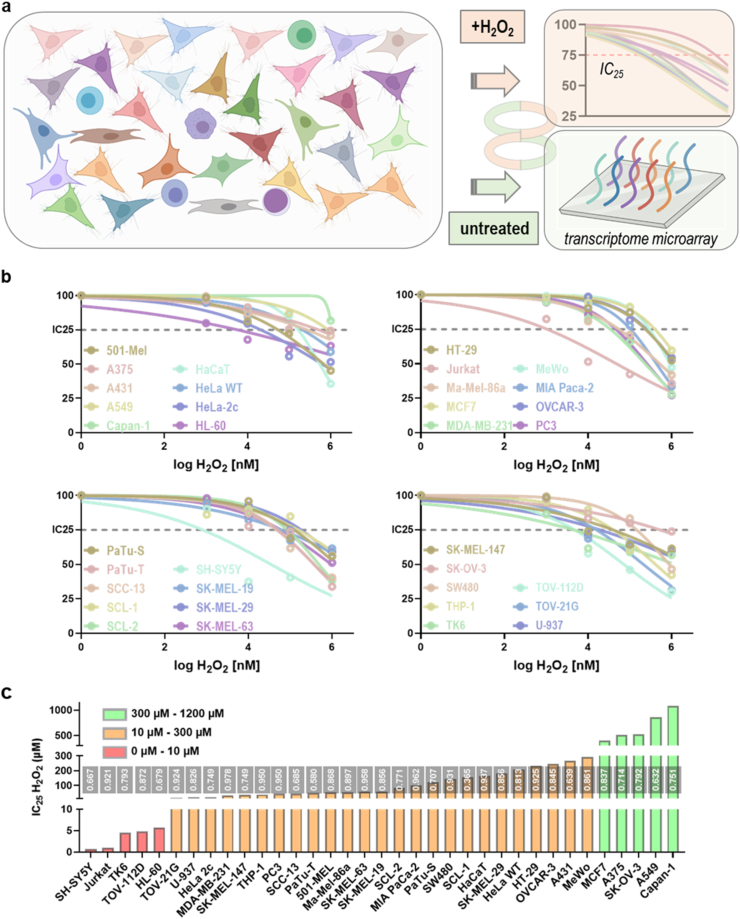
**Study overview and IC**_**25**_**determination.** (**a**) 35 cell lines were cultured and RNA was collected from untreated cells, while in parallel, from cultured cells, the H_2_O_2_ IC_25_ was determined, and both data sets were correlated; (**b**) log-transformed H_2_O_2_ concentrations plotted against normalized metabolic activity rates of cells 24 h after exposure to H_2_O_2_, and non-linear regression analysis to identify the IC_25_ for each cell line; (**c**) waterfall plot of all cell lines sorted for IC_25_ and color-coded for different sensitivity ranges. Data are from three independent experiments. Grey boxes indicate the goodness of fit (R^2^) for each cell line. Fig. 1a created with biorender.com. (For interpretation of the references to color in this figure legend, the reader is referred to the Web version of this article.)

This study aimed to identify genes associated with the sensitivity and resistance of H_2_O_2_-induced cellular demise across 35 cell lines, with 34 of them being tumor cell lines (Fig. 1a). To this end, in-house transcriptomic profiling of the untreated cell lines was performed. In parallel, the inhibitory concentration (IC_25_) of H_2_O_2_ was determined for each cell line. Both datasets were correlated to identify genes with a potentially predictive value on oxidative stress sensitivity. The IC_25_ values were obtained by measuring the metabolic activity of each of the cell types 24 h after exposure to different concentrations of H_2_O_2_. Non-linear curve fitting was employed. Except for the Capan-1 cell lines, all cell lines had at least one H_2_O_2_ concentration decreasing metabolic activity by at least 25% to generate valid IC_25_ values (Fig. 1b). The results are shown in a waterfall plot, indicating an differences of more than 100-fold between the most and least sensitive cell lines (Fig. 1c). The goodness of fit (R^2^; grey boxes) was high (>0.6) for all cell lines except PaTu-T and SCL-1, where a higher degree of uncertainty of the IC_25_ can be expected.

**Fig. 2:**
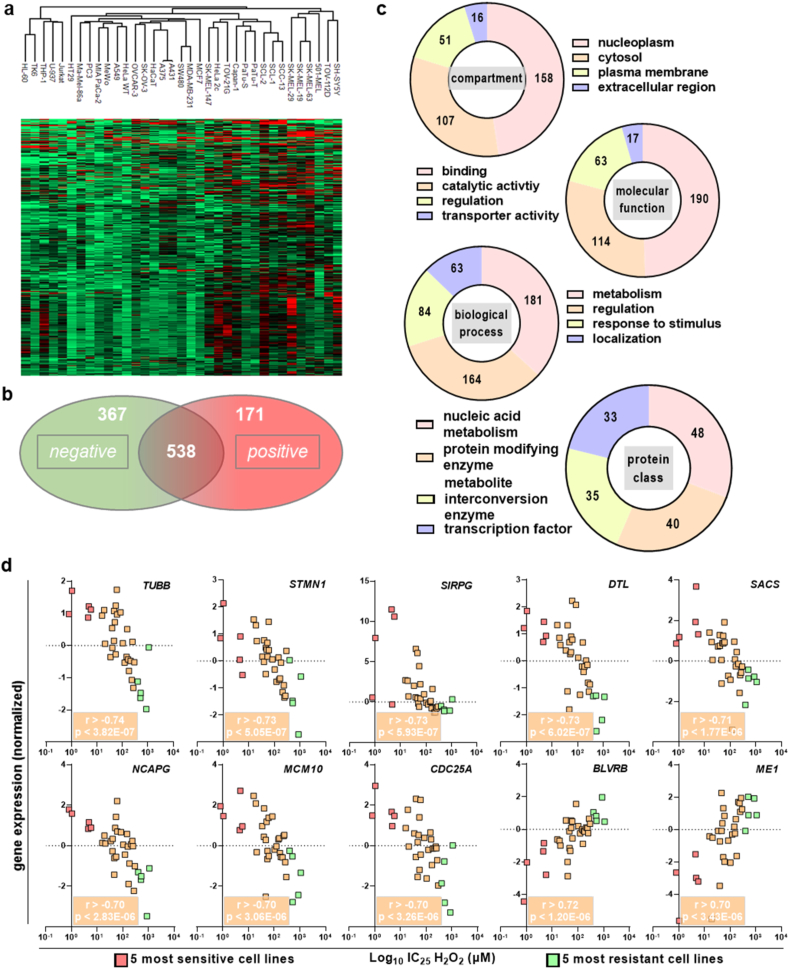
**Transcriptomic analysis.** (**a**) hierarchical clustering of normalized gene expression across 35 cell lines confirms relationships, e.g., between the SK-MEL melanoma cell lines (right end) and the five leukemia cell lines (left) clustering together; (**b**) the number of genes positively or negatively correlating with IC_25_ values for p < 0.01 and r > 0.50 or r < −0.50, respectively; (**c**) display of top four classes among target compartment, molecular function, biological process, and protein class for the significantly correlating genes (538) with the values indicating the number of genes per category as per gene ontology (PANTHER) analysis; (**d**) normalized gene expression of the transcriptomic chip data of the top 10 target genes sorted for IC_25_ values and indicating the five most sensitive and resistant cell lines showing high correlation (r) values and statistical significance (yellow boxes). (For interpretation of the references to color in this figure legend, the reader is referred to the Web version of this article.)

Transcriptional analysis of baseline gene expression and subsequent hierarchical clustering indicating close relationships for cellular origins. For instance, the leukemia cell lines TK6, HL-60, U-937, Jurkat, and THP-1 as well as the melanoma cell lines SK-MEL-19, SK-MEL,63- and SK-MEL-29 clustered together (Fig. 2a). Such melanoma and leukemia cell line clusters were confirmed using principal component analysis (Fig. S1). Correlation analysis of transcriptional expression and H_2_O_2_ IC_25_ values revealed 171 and 367 to correlate positively (Table 1) or negatively (Table 2), respectively (Fig. 2b). These genes were further segmented based on gene ontology analysis for compartment, molecular function, biological process, and protein class (Fig. 2c). The top categories were nucleoplasmic localization, protein binding activity, metabolic processes, and nucleic acid metabolism, suggesting many of the genes identified to be involved in cellular growth and cell cycle progression (Table 3). Relative intensities of significant genes across all cell lines were shown for positively (Fig. S2) and negatively (Fig. S3) correlating (r > 0.50/r < −0.50) targets. The top 10 genes correlating with sensitivity to H_2_O_2_-mediated metabolic activity reduction were identified and graphed for each cell line individually (Fig. 2d).

**Table 1:** Positively correlating genes. Genes, p-values, and Spearman r of genes positively correlating with resistance to H_2_O_2_-mediated cellular demise with an r > 0.50.

gene	p-value	r	gene	p-value	r	gene	p-value	r
BLVRB	1.20E-06	0.72	ME1	3.43E-06	0.70	POLD4	3.61E-06	0.69
SLC35A3	5.96E-06	0.68	KIAA1522	6.49E-06	0.68	AREG	7.78E-06	0.68
CFB	1.09E-05	0.67	PTGR1	1.36E-05	0.66	CAPZA2	1.49E-05	0.66
PERP	1.54E-05	0.66	ANXA9	1.56E-05	0.66	MOCOS	1.70E-05	0.66
TSPAN31	1.97E-05	0.65	TPD52L1	2.55E-05	0.65	TOM1L1	3.12E-05	0.64
OR6K2	3.39E-05	0.64	MTFR1	4.01E-05	0.64	RAB26	5.06E-05	0.63
FTHL17	5.76E-05	0.63	MOS	5.99E-05	0.62	CDNF	7.01E-05	0.62
HSBP1L1	8.42E-05	0.62	TC2N	8.50E-05	0.61	UGDH	8.50E-05	0.61
ATP8B1	1.03E-04	0.61	ELOVL7	1.10E-04	0.61	PRSS22	1.10E-04	0.61
CCDC104	1.14E-04	0.61	UPK3B	1.17E-04	0.61	ZFAND1	1.19E-04	0.60
RNF103	1.22E-04	0.60	LCN2	1.39E-04	0.60	OSGIN1	1.44E-04	0.60
GATA6	1.44E-04	0.60	EXOC3L4	1.52E-04	0.60	TMEM54	1.54E-04	0.60
CEBPB	1.68E-04	0.59	CREG1	1.71E-04	0.59	UBXN2B	1.95E-04	0.59
POM121L12	2.02E-04	0.59	TSPAN1	2.13E-04	0.59	SEL1L3	2.15E-04	0.59
AQP11	2.15E-04	0.59	EPB41L4B	2.17E-04	0.59	GRTP1	2.24E-04	0.58
UGT1A6	2.36E-04	0.58	TATDN1	2.36E-04	0.58	FAM164C	2.80E-04	0.58
CCDC122	2.92E-04	0.58	DCTN1	3.02E-04	0.58	KRT18	3.02E-04	0.58
ECI1	3.07E-04	0.57	CTNND1	3.31E-04	0.57	KRTAP10	3.39E-04	0.57
EREG	3.62E-04	0.57	VRK2	3.77E-04	0.57	TMC4	3.81E-04	0.57
TMEM105	3.87E-04	0.57	BCL6	4.09E-04	0.56	FXYD3	4.30E-04	0.56
DSP	4.37E-04	0.56	PKP3	4.44E-04	0.56	CDH1	4.51E-04	0.56
UBE2H	4.55E-04	0.56	AHNAK	4.58E-04	0.56	ASPH	4.58E-04	0.56
YIF1B	5.04E-04	0.56	PARD6B	5.08E-04	0.56	F2RL1	5.24E-04	0.56
PRSS16	5.94E-04	0.55	CLIC3	6.27E-04	0.55	MAL2	6.42E-04	0.55
BCL3	6.52E-04	0.55	KLF5	6.52E-04	0.55	PCDH1	6.52E-04	0.55
FAM118A	6.88E-04	0.55	KBTBD13	7.04E-04	0.55	FA2H	7.14E-04	0.54
CDS1	7.14E-04	0.54	LGALS3	7.20E-04	0.54	MALL	7.31E-04	0.54
TMC5	7.48E-04	0.54	ASB16	7.48E-04	0.54	SYT7	7.59E-04	0.54
MTUS1	7.65E-04	0.54	CHMP4C	7.71E-04	0.54	ITGB4	7.82E-04	0.54
CLDN23	7.88E-04	0.54	IGF1R	9.69E-04	0.54	SCNN1A	8.43E-04	0.54
PLLP	8.56E-04	0.54	MARVELD2	8.56E-04	0.54	NHLRC1	8.82E-04	0.54
RAB17	8.82E-04	0.54	FANK1	8.82E-04	0.54	TTC39A	9.01E-04	0.54
BHLHE41	9.01E-04	0.54	S100A14	9.01E-04	0.54	EPCAM	9.01E-04	0.54
CXCL16	9.28E-04	0.54	KIAA1244	9.35E-04	0.53	UGT1A8	9.63E-04	0.53
PLA2G10	9.63E-04	0.53	TMEM125	1.04E-03	0.53	GOLGA5	1.11E-03	0.53
IL28A	1.13E-03	0.53	MYO5B	1.15E-03	0.53	CAV2	1.15E-03	0.53
BOD1	1.16E-03	0.53	HM13	1.19E-03	0.53	PDGFB	1.42E-03	0.53
FOXA1	1.21E-03	0.52	MYEOV	1.21E-03	0.52	NXPH4	1.27E-03	0.52
SH3D19	1.31E-03	0.52	PPFIA1	1.32E-03	0.52	LHX1	1.38E-03	0.52
PHGR1	1.40E-03	0.52	MBNL2	1.41E-03	0.52	EPB41L5	1.46E-03	0.52
FAM179B	1.47E-03	0.52	FAM83H	1.47E-03	0.52	CSNK1A1L	1.49E-03	0.52
MYNN	1.56E-03	0.51	IQCK	1.60E-03	0.51	VTN	1.60E-03	0.51
TMEM61	1.62E-03	0.51	SERPINA3	1.62E-03	0.51	EPS8L2	1.63E-03	0.51
CD8A	1.63E-03	0.51	BTBD16	1.63E-03	0.51	CALCOCO2	1.65E-03	0.51
BUD31	1.68E-03	0.51	MUC1	1.69E-03	0.51	MTA3	1.69E-03	0.51
ADPRHL1	1.70E-03	0.51	KLF4	1.71E-03	0.51	ANKRD2	1.71E-03	0.51
RNLS	1.72E-03	0.51	YES1	1.76E-03	0.51	METRNL	2.10E-03	0.51
ZNF572	1.79E-03	0.51	KRTAP6-2	1.81E-03	0.51	KRT8	1.82E-03	0.51
GDF15	1.83E-03	0.51	PROM2	1.83E-03	0.51	MET	1.87E-03	0.51
DNAJB9	1.88E-03	0.51	MECOM	1.89E-03	0.51	GLCE	1.91E-03	0.51
NTF4	1.92E-03	0.51	KRT19	2.00E-03	0.50	RHOD	2.01E-03	0.50
A4GALT	2.01E-03	0.50	GJB2	2.03E-03	0.50	RBCK1	2.03E-03	0.50
PTK6	2.39E-03	0.50	C5orf32	2.04E-03	0.50	SOCS6	2.10E-03	0.50
NFIL3	2.12E-03	0.50	PLEKHH2	2.15E-03	0.50	GABRE	2.21E-03	0.50
TSPAN15	2.21E-03	0.50	LONP2	2.31E-03	0.50	EFNA1	2.31E-03	0.50
DEGS2	2.31E-03	0.50	SYBU	2.36E-03	0.50	INADL	2.36E-03	0.50
CDC91	2.38E-03	0.50	DYNC2LI1	2.41E-03	0.50	NRIP1	2.44E-03	0.50
ADAM9	2.46E-03	0.50	/	/	/	/	/	/

**Table 2:** Negatively correlating genes. Genes, p-values, and Spearman r of genes negatively correlating with resistance to H_2_O_2_-mediated cellular demise with an r < −0.50.

gene	p-value	r	gene	p-value	r	gene	p-value	r
TUBB	3.82E-07	−0.74	STMN1	5.50E-07	−0.73	SIRPG	5.93E-07	−0.73
DTL	6.02E-07	−0.73	SACS	1.77E-06	−0.71	NCAPG	2.83E-06	−0.70
MCM10	3.06E-06	−0.70	CDC25A	3.26E-06	−0.70	CPXM1	3.76E-06	−0.69
H2BFM	3.95E-06	−0.69	NRGN	1.20E-05	−0.67	GTSE1	1.38E-05	−0.66
NEUROG2	1.52E-05	−0.66	LIMD2	1.68E-05	−0.66	CCDC28B	1.74E-05	−0.66
NASP	1.92E-05	−0.66	PSMC2	1.99E-05	−0.65	MCM2	2.37E-05	−0.65
TACC3	2.37E-05	−0.65	BLM	2.75E-05	−0.65	CDCA8	2.81E-05	−0.65
MLF1IP	2.81E-05	−0.65	RNASE6	2.81E-05	−0.65	GPR63	2.87E-05	−0.65
MKI67	3.09E-05	−0.64	RFTN1	3.29E-05	−0.64	ZNF124	3.96E-05	−0.64
XRCC2	4.26E-05	−0.63	CCNA2	4.35E-05	−0.63	PHF19	4.39E-05	−0.63
HIC1	5.43E-05	−0.63	FBXO5	5.53E-05	−0.63	OSM	5.59E-05	−0.63
HNRNPU	6.23E-05	−0.62	TAGAP	6.74E-05	−0.62	ORC6	6.94E-05	−0.62
RNASE2	7.22E-05	−0.62	UBE2T	7.29E-05	−0.62	MCART6	7.95E-05	−0.62
BTK	8.10E-05	−0.62	ZNF692	8.26E-05	−0.62	MCM5	8.58E-05	−0.61
NCAPD2	8.91E-05	−0.61	CXXC1	8.91E-05	−0.61	EIF4E2	9.00E-05	−0.61
CORO1A	9.98E-05	−0.61	FANCA	9.98E-05	−0.61	KIAA0586	9.98E-05	−0.61
UHRF1	1.08E-04	−0.61	PTPN22	1.09E-04	−0.61	MIS18A	1.12E-04	−0.61
ELAVL1	1.14E-04	−0.61	HELLS	1.15E-04	−0.61	ESPL1	1.17E-04	−0.61
ITPRIPL1	1.24E-04	−0.60	SAP30	1.25E-04	−0.60	NUP62	1.28E-04	−0.60
CCDC77	1.35E-04	−0.60	EME1	1.37E-04	−0.60	PLEKHO1	1.39E-04	−0.60
FMNL3	1.39E-04	−0.60	DIAPH3	1.42E-04	−0.60	VRK1	1.52E-04	−0.60
DBF4B	1.52E-04	−0.60	KCNA6	1.62E-04	−0.60	DNA2	1.64E-04	−0.59
PTGIR	1.69E-04	−0.59	ZNF100	1.73E-04	−0.59	ORC1	1.74E-04	−0.59
TYMS	1.82E-04	−0.59	SRRT	1.88E-04	−0.59	KIF11	1.88E-04	−0.59
FHOD1	1.92E-04	−0.59	IL12RB1	1.93E-04	−0.59	HIVEP3	1.95E-04	−0.59
KIF2C	1.99E-04	−0.59	TNFAIP8L2	2.02E-04	−0.59	KIF14	2.07E-04	−0.59
CLSPN	2.07E-04	−0.59	CEP85	2.07E-04	−0.59	RAD54L	2.09E-04	−0.59
JAM2	2.15E-04	−0.59	RUFY2	2.19E-04	−0.59	C1orf187	2.20E-04	−0.59
RAD54L2	2.22E-04	−0.59	FANCD2	2.24E-04	−0.58	KIF15	2.24E-04	−0.58
PDXP	2.24E-04	−0.58	E2F1	2.26E-04	−0.58	GTPBP3	2.36E-04	−0.58
MAP3K4	2.40E-04	−0.58	DNAJB5	2.42E-04	−0.58	CASC5	2.49E-04	−0.58
ZADH2	2.51E-04	−0.58	SETMAR	2.57E-04	−0.58	SLA	2.71E-04	−0.58
WASF1	2.71E-04	−0.58	SLA	2.71E-04	−0.58	BIRC5	2.71E-04	−0.58
TNFRSF8	2.78E-04	−0.58	WDR76	2.94E-04	−0.58	CCNF	2.97E-04	−0.58
TSPYL4	3.02E-04	−0.58	TDP1	3.05E-04	−0.57	CD244	3.05E-04	−0.57
MEX3B	3.07E-04	−0.57	EVI2A	3.10E-04	−0.57	CENPE	3.12E-04	−0.57
HNRNPA3	3.15E-04	−0.57	EBLN2	3.15E-04	−0.57	PHRF1	3.20E-04	−0.57
BUB1B	3.34E-04	−0.57	NCKAP1L	3.36E-04	−0.57	CENPJ	3.54E-04	−0.57
NKG7	3.56E-04	−0.57	FADS2	4.55E-04	−0.57	MYLK2	3.74E-04	−0.57
MCM3	3.74E-04	−0.57	WTAP	3.74E-04	−0.57	POLQ	3.81E-04	−0.57
PTPRC	3.81E-04	−0.57	FDXACB1	3.84E-04	−0.57	E2F2	3.90E-04	−0.57
MCM6	4.30E-04	−0.56	PURG	4.33E-04	−0.56	TROAP	4.33E-04	−0.56
PIF1	4.40E-04	−0.56	RFC3	4.58E-04	−0.56	SMAP2	4.62E-04	−0.56
CHAF1A	4.62E-04	−0.56	SMAP2	4.62E-04	−0.56	FCHSD2	4.66E-04	−0.56
SIRPB1	4.73E-04	−0.56	ABCD2	4.77E-04	−0.56	DDX39A	4.77E-04	−0.56
OPRL1	4.84E-04	−0.56	KIAA1661	4.88E-04	−0.56	ST8SIA5	5.00E-04	−0.56
LMNB1	5.04E-04	−0.56	VPRBP	5.04E-04	−0.56	MASTL	5.08E-04	−0.56
DNMT1	5.24E-04	−0.56	RBM15	5.28E-04	−0.56	ZNF878	5.33E-04	−0.56
GMNN	5.37E-04	−0.56	MAPRE2	5.37E-04	−0.56	ILF3	5.45E-04	−0.55
SRSF1	5.54E-04	−0.55	PPP2R3B	5.54E-04	−0.55	AP1M1	5.63E-04	−0.55
MPO	5.63E-04	−0.55	RRM1	5.67E-04	−0.55	SPC25	5.67E-04	−0.55
SPC25	5.67E-04	−0.55	FERMT3	5.71E-04	−0.55	HVCN1	5.80E-04	−0.55
WDR62	5.80E-04	−0.55	POLE2	5.85E-04	−0.55	CEP44	5.85E-04	−0.55
ARID3B	5.90E-04	−0.55	POLA1	5.90E-04	−0.55	SLC9B2	5.94E-04	−0.55
GTF3C3	5.94E-04	−0.55	POC1A	5.99E-04	−0.55	SEZ6	6.03E-04	−0.55
MXD3	6.22E-04	−0.55	LIG1	7.61E-04	−0.55	LIG1	7.61E-04	−0.55
ZNF519	6.32E-04	−0.55	ADRBK2	6.32E-04	−0.55	ZNF589	6.42E-04	−0.55
CENPO	6.57E-04	−0.55	BCOR	6.62E-04	−0.55	ZCCHC18	6.67E-04	−0.55
HNRNPD	6.82E-04	−0.55	SUV39H1	6.82E-04	−0.55	GUCY1A3	6.98E-04	−0.55
TPM3	7.04E-04	−0.55	PASK	7.14E-04	−0.54	IQSEC3	7.14E-04	−0.54
ANKRD36B	7.14E-04	−0.54	RASSF5	7.25E-04	−0.54	GAMT	7.31E-04	−0.54
GPR183	8.88E-04	−0.54	PDSS1	7.36E-04	−0.54	SRSF2	7.48E-04	−0.54
LAIR2	7.48E-04	−0.54	EVI2B	7.48E-04	−0.54	ARHGAP19	7.53E-04	−0.54
CEBPE	7.59E-04	−0.54	ACAT2	7.65E-04	−0.54	ACAT2	7.65E-04	−0.54
MYBL2	9.24E-04	−0.54	RPL27A	7.76E-04	−0.54	SFMBT2	7.94E-04	−0.54
HNRNPA2B1	8.00E-04	−0.54	IL10RA	8.06E-04	−0.54	CFP	8.06E-04	−0.54
SLAMF6	8.18E-04	−0.54	P2RY10	8.43E-04	−0.54	GPR174	8.43E-04	−0.54
CSRNP3	8.43E-04	−0.54	KIF26B	8.56E-04	−0.54	GINS3	8.62E-04	−0.54
ARHGAP11A	8.69E-04	−0.54	SGOL2	8.75E-04	−0.54	ZNF407	8.95E-04	−0.54
ATP6V1G2	9.01E-04	−0.54	APOBEC3G	9.01E-04	−0.54	C12orf24	9.35E-04	−0.53
PVRIG	9.35E-04	−0.53	ASF1B	9.42E-04	−0.53	DCAF15	9.63E-04	−0.53
NARF	9.63E-04	−0.53	SAMSN1	9.70E-04	−0.53	AGPAT5	9.92E-04	−0.53
KIFC1	9.92E-04	−0.53	TRAF3IP3	9.99E-04	−0.53	FANCB	1.01E-03	−0.53
SPC24	1.01E-03	−0.53	GIMAP8	1.03E-03	−0.53	SLC4A8	1.04E-03	−0.53
CMTM3	1.05E-03	−0.53	RRM2	1.06E-03	−0.53	VKORC1	1.06E-03	−0.53
CCDC136	1.07E-03	−0.53	ZWINT	1.09E-03	−0.53	MTR	1.09E-03	−0.53
SOX12	1.11E-03	−0.53	SOX12	1.11E-03	−0.53	TXLNB	1.11E-03	−0.53
ZNF273	1.11E-03	−0.53	GCFC1	1.11E-03	−0.53	ZKSCAN4	1.11E-03	−0.53
DACT3	1.12E-03	−0.53	LILRB4	1.12E-03	−0.53	AGER	1.13E-03	−0.53
FRYL	1.13E-03	−0.53	SLC25A19	1.15E-03	−0.53	GNGT2	1.15E-03	−0.53
TSHZ1	1.15E-03	−0.53	CBX2	1.17E-03	−0.53	EMILIN1	1.18E-03	−0.53
TLE4	1.18E-03	−0.53	ARHGAP9	1.20E-03	−0.53	MYBPC2	1.21E-03	−0.52
CCNC	1.21E-03	−0.52	RGS1	1.22E-03	−0.52	DOK3	1.22E-03	−0.52
PLK4	1.25E-03	−0.52	RBM14	1.29E-03	−0.52	HNRNPL	1.29E-03	−0.52
RGS9	1.29E-03	−0.52	PRKCB	1.30E-03	−0.52	MS4A7	1.30E-03	−0.52
CTSC	1.31E-03	−0.52	ARHGAP33	1.31E-03	−0.52	CEP135	1.35E-03	−0.52
CCL3	1.38E-03	−0.52	CBX5	1.39E-03	−0.52	PMS1	1.44E-03	−0.52
WRAP53	1.47E-03	−0.52	BMP2K	1.48E-03	−0.52	VASH2	1.48E-03	−0.52
FANCG	1.49E-03	−0.52	SAFB	1.50E-03	−0.52	LILRB1	1.52E-03	−0.52
ANKRD20A1	1.52E-03	−0.52	ORAI2	1.54E-03	−0.52	ANKRD32	1.54E-03	−0.52
WDR82	1.55E-03	−0.51	CD37	1.55E-03	−0.51	ACAP1	1.57E-03	−0.51
PLK1	1.57E-03	−0.51	FBXO43	1.61E-03	−0.51	DOK2	1.61E-03	−0.51
SLC6A13	1.62E-03	−0.51	SF3B1	1.64E-03	−0.51	FUS	1.64E-03	−0.51
CYTH1	1.64E-03	−0.51	HMGB2	1.64E-03	−0.51	KRI1	1.65E-03	−0.51
FANCM	1.65E-03	−0.51	NLRP13	1.65E-03	−0.51	METTL3	1.66E-03	−0.51
ADORA2A	1.68E-03	−0.51	DOT1L	1.69E-03	−0.51	BUB3	1.69E-03	−0.51
SRSF3	1.69E-03	−0.51	CAPN6	1.70E-03	−0.51	UNC13C	1.71E-03	−0.51
PARVG	1.72E-03	−0.51	ESPNL	1.73E-03	−0.51	LUC7L3	1.75E-03	−0.51
PKHD1L1	1.75E-03	−0.51	PNN	1.75E-03	−0.51	SMARCC1	1.75E-03	−0.51
TRAIP	1.77E-03	−0.51	BARD1	1.77E-03	−0.51	RAG2	1.78E-03	−0.51
LSM2	1.79E-03	−0.51	GNAI2	1.79E-03	−0.51	CCDC75	1.81E-03	−0.51
SRSF11	1.81E-03	−0.51	CNTROB	1.81E-03	−0.51	HJURP	1.83E-03	−0.51
EXO1	1.83E-03	−0.51	MND1	1.84E-03	−0.51	GEMIN4	1.84E-03	−0.51
TERT	1.84E-03	−0.51	PTPN7	1.84E-03	−0.51	CXCR4	1.87E-03	−0.51
ACIN1	1.93E-03	−0.51	LEPROT	1.93E-03	−0.51	DNASE1L3	1.96E-03	−0.51
ING3	1.97E-03	−0.51	TTN	1.99E-03	−0.50	PRPS1	1.99E-03	−0.50
MYO1G	2.00E-03	−0.50	FRMD4A	2.03E-03	−0.50	INCENP	2.03E-03	−0.50
LCORL	2.03E-03	−0.50	ZC4H2	2.04E-03	−0.50	WDR6	2.05E-03	−0.50
GPSM3	2.07E-03	−0.50	XAB2	2.08E-03	−0.50	SASH3	2.10E-03	−0.50
ANXA6	2.11E-03	−0.50	SAP25	2.11E-03	−0.50	UPB1	2.12E-03	−0.50
LIX1L	2.17E-03	−0.50	CDC42	2.17E-03	−0.50	TNFAIP8L1	2.18E-03	−0.50
NRXN2	2.18E-03	−0.50	CSTF3	2.20E-03	−0.50	IL16	2.21E-03	−0.50
CKS2	2.21E-03	−0.50	RBM10	2.21E-03	−0.50	PSTPIP1	2.22E-03	−0.50
E2F7	2.22E-03	−0.50	MICAL1	2.22E-03	−0.50	KIAA0748	2.22E-03	−0.50
KBTBD8	2.24E-03	−0.50	FGFR1	2.25E-03	−0.50	KIF22	2.25E-03	−0.50
PARP1	2.25E-03	−0.50	MCM7	2.28E-03	−0.50	SPAG6	2.28E-03	−0.50
SEPT03	2.28E-03	−0.50	UNC79	2.28E-03	−0.50	MYO18B	2.31E-03	−0.50
ZKSCAN3	2.35E-03	−0.50	DHX30	2.35E-03	−0.50	EGFEM1P	2.38E-03	−0.50
GCDH	2.38E-03	−0.50	NLRP12	2.38E-03	−0.50	LAX1	2.39E-03	−0.50
CD53	2.39E-03	−0.50	DNAJC9	2.39E-03	−0.50	KSR1	2.41E-03	−0.50
CD69	2.41E-03	−0.50	HGF	2.44E-03	−0.50	CACNB2	2.44E-03	−0.50
AGAP2	2.47E-03	−0.50	NKTR	2.49E-03	−0.50	/	/	/

**Table 3:** Pathway enrichment. Functional category, enrichment false discovery rate (FDR), and the number of pathway-related genes of all significantly correlating genes with an r > 0.50 or r < −0.50.

Functional category	Enrichment FDR	Number of genes (r > 0.50)
Mitotic cell cycle process	1.2E-22	83
Cell cycle	2.3E-22	124
Mitotic cell cycle	5.4E-22	88
Cell cycle process	1.5E-20	102
DNA replication	2.5E-15	38
Chromosome organization	1.9E-12	79
DNA-dependent DNA replication	2.8E-12	26
Mitotic nuclear division	2.8E-12	35
Nuclear division	3.2E-12	43
Organelle organization	1.2E-11	169

**Fig. 3:**
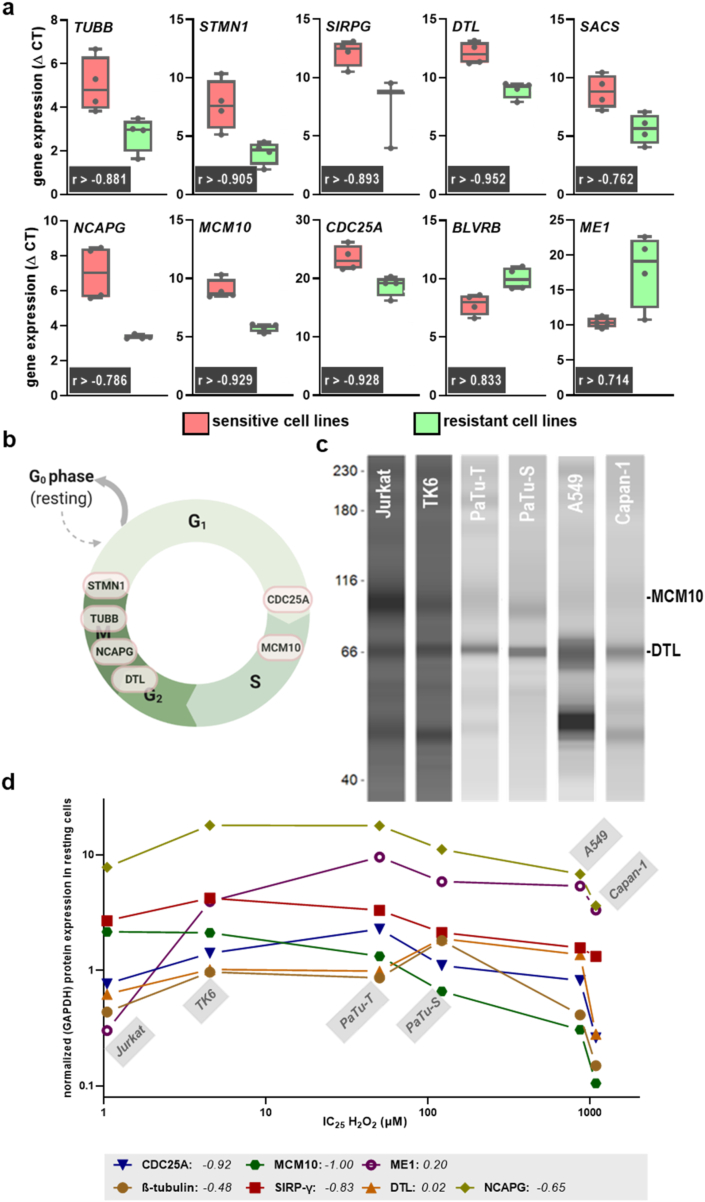
**Top 10 target validation.** (**a**) validation of target genes using qPCR in eight selected cell lines (four sensitives, four resistant) from two experiments shown as box plots and fully confirming findings by transcriptomic analysis (grey boxes, significant and high correlation between expression and H_2_O_2_ IC_25_); (**b**) graphical display of six of the ten target genes being directly involved in cell cycle progression; (**c**) representative Western blot images from capillary-based gel electrophoresis (WES) for six cell lines and indicating two representative targets; (**d**) quantitative protein expression from three experiments after normalization against GAPDH and plotted against the predetermined H_2_O_2_ IC_25_, grey box indicating Spearman correlation. Fig. 3b created with biorender.com.

Subsequently, the top 10 genes were validated using qPCR in a selected number of cell lines (Fig. 3a). Strikingly, for all genes identified in the transcriptomic analysis, a significant and high correlation could be identified by manual single target qPCR analysis. This underlined the stringency and validity of our microarray experiments. The targets' co-expression signatures were subsequently analyzed and six of the ten targets clustered in a single network while the other four showed no direct interrelationship (Fig. S4). It was identified that these six targets are directly related to cell cycle progression (Fig. 3b). Next, the protein expression of these six targets and SIPRG was quantified in six cell lines using Western blot (Fig. 3c). Relative protein expression was plotted against the predetermined H_2_O_2_ IC_25_ values (Fig. 3d). The overall correlation was confirmed on the protein level, with DTL lacking a negative correlation and ME1 having a lower positive correlation as suggested by gene expression. Concerning the top 10 genes significantly and strongly correlating with H_2_O_2_-mediated cellular demise, six of the targets are entangled in cell cycle progression. Thus, we compared serum-starved against serum-supplemented cultures but found only an heterogenous change in baseline metabolic activity across sensitive and resistant cell lines (Fig. S5a), while cell cycle arrest was found in four cell lines tested (Fig. S5b). Upon H_2_O_2_ challenge, seven out of ten cell lines tested showed enhanced sensitivity (fold changes >1.0) to cellular demise (Fig. S5c) with no clear picture emerging towards their previously identified sensitivity or resistance to H_2_O_2_. qPCR analysis of five genes was done and normalized to GAPDH shown as starved over serum expression (Fig. S5d). NCAPG (Jurkat) and TUBB and CDC25A (TK6) decreased more than two-fold, indicating starvation-induced cell cycle arrest. In PaTu-S, DTL and NCAPG were similarly decreased while CDC25A was markedly increased. A549 cells showed a pronounced upregulation of cell cycle-related genes. Altogether, starvation experiments were not able to explain the sensitivity or resistance to H_2_O_2_.

**Fig. 4:**
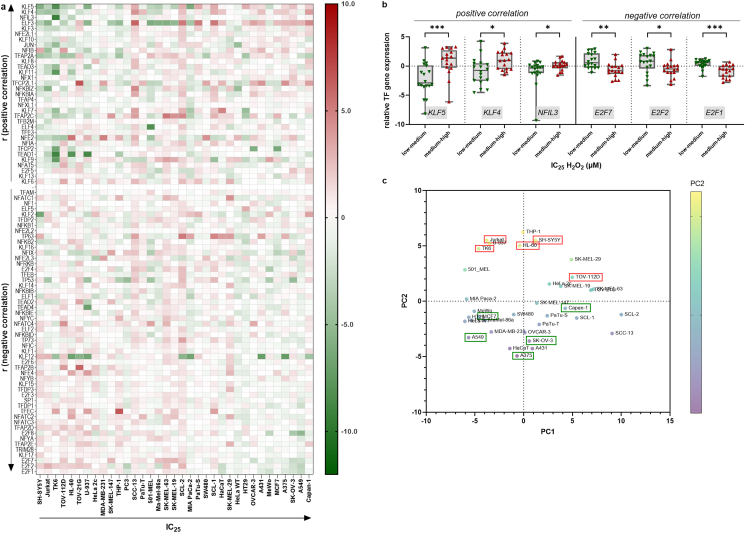
**Transcription factor gene expression.** (**a**) heatmap of relative transcription factor expression normalized to the mean expression of each gene across all 35 cell lines sorted for positive and negative correlation (r) against the H_2_O_2_ IC_25_ of each cell line; (**b**) transcription factors with Spearman r > 0.5 (left) and <-0.5 (right) and their relative expression graphed for cell lines 1–18 with low to medium H_2_O_2_ IC_25_ values and cell lines 19–35 with medium to high H_2_O_2_ IC_25_ values. Statistical analysis was done using Mann-Whitney test with p < 0.05 (*), p < 0.01 (**), and p < 0.001 (***); (**c**) principal component (PC) analysis of the relative expression of the transcription factors of (a) across all cell lines identifies PC2 to segregate between H_2_O_2_-sensitive (top, red boxes) and resistant (bottom, green boxes) cell lines. (For interpretation of the references to color in this figure legend, the reader is referred to the Web version of this article.)

To provide hints towards the mechanism of actions, the expression of several transcription factors (TF) was correlated against the H_2_O_2_ IC_25_ of cells (Fig. 4a). Six TF showed a good (Spearman r > 0.50 or < -0.50) positive (KLF5, KLF4, and NFIL3) and negative (E2F1, E2F2, and E2F7) correlation (Table S1). Cumulative analysis of these TF across 18 more sensitive vs. 17 more resistant cell lines showed their relative expression distribution to be significantly different (Fig. 4b). Principal compenent (PC) analysis of all TF across the 35 cell lines investigated revealed PC2 to be predictive for the five most sensitive (red boxes) and resistant (green boxes) cell lines (Fig. 4c). These results suggested an involvement of TF in H_2_O_2_-mediated toxicity albeit it should be stressed that their relatively enhanced or decreased expression was still heterogenous across the cell lines (Fig. 4a), and their correlation was good but not high.

Finally, the correlation of genes associated with ROS/RNS production, redox signaling, and antioxidant defense was analyzed in our data set (Table 4). No gene showed a high correlation but a good correlation (Spearman r > 0.40 or < -0.40) was identified for GPX2 (Glutathione peroxidase 2), NOXA1 (NADPH oxidase activator 1), and GSTO2 (Glutathione S-transferase omega-2) (positive correlation) and NOS2 (Nitric oxide synthase 2), GPX7 (Glutathione peroxidase 2), and GSTCD (glutathione S-transferase A7) (negative correlation). Again, their relative expression was overall heterogenous across all cell lines investigated (Fig. S6). For instance, the most resistant cell lines Capan-1 and A549 showed markedly enhanced levels of GPX2, while the third and fourth most resistant cell lines SK-OV-3 and A375 identified with a below-average expression.

**Table 4:** Correlation of ROS-related proteins. Given are genes and protein names of ROS-related and antioxidant proteins positively (left) and negatively (right) correlating to H_2_O_2_ IC_25_ values. Genes with Spearman r correlations of >0.40 and <-0.40 are marked in grey.

Gene	Protein	r (+)	Gene	Protein	r (−)
GPX2	glutathione peroxidase 2	0.44	NOS2	nitric oxide synthase, inducible	−0.45
NOXA1	NADPH oxidase activator 1, NOX activator 1	0.42	GPX7	glutathione peroxidase 7	−0.41
GSTO2	glutathione S-transferase omega 2	0.41	GSTCD	glutathione S-transferase A7	−0.40
NOXO1	NADPH oxidase organizer 1	0.26	GSTA7P	glutathione S-transferase CD	−0.39
TXNDC5	thioredoxin domain-containing protein 5	0.26	GSTM5	glutathione S-transferase Mu 5	−0.35
GSTZ1	glutathione S-transferase zeta 1	0.26	GSTM2	glutathione S-transferase Mu 2	−0.33
ENOX2	ecto-NOX disulfide-thiol exchanger 2	0.21	GPX6	glutathione peroxidase 6	−0.33
GSTT1	glutathione S-transferase T1	0.21	TXN2	thioredoxin 2	−0.30
TXN	thioredoxin	0.20	GSTM2P1	glutathione S-transferase Mu2P1	−0.29
SOD2	superoxide dismutase 2	0.18	GSTTP1	glutathione S-transferase TP1	−0.27
GSTK1	glutathione S-transferase kappa 1	0.17	TXNDC3	thioredoxin domain-containing protein 3	−0.26
GSTA5	glutathione S-transferase A7P	0.17	NOX1	NADPH oxidase 1	−0.25
PRDX5	peroxiredoxine 5	0.17	GSTTP2	glutathione S-transferase TP2	−0.24
TXNIP	thioredoxin interacting protein	0.16	NOX3	NADPH oxidase 3	−0.24
GPX8	glutathione peroxidase 8	0.16	NOX4	NADPH oxidase 4	−0.23
GSTT2	glutathione S-transferase T2	0.15	NOS3	nitric oxide synthase, endothelial	−0.22
PRDX4	peroxiredoxine 4	0.15	TXNDC16	thioredoxin domain-containing protein 16	−0.20
TXNL4B	thioredoxin-like protein 4B	0.14	TXNL4A	thioredoxin-like protein 4A	−0.20
GPX4	glutathione peroxidase 4	0.14	NOS1	nitric oxide synthase	−0.18
TXNDC9	thioredoxin domain-containing protein9	0.14	TXNDC8	thioredoxin domain-containing protein 8	−0.17
TXNDC12	thioredoxin domain-containing protein 12	0.12	PRDX2	peroxiredoxine 2	−0.16
GSTP1	glutathione S-transferase P1	0.12	SOD1	superoxide dismutase 1	−0.14
DUOX2	Dual oxidase 2	0.12	TXNL1	thioredoxin-like protein 1	−0.14
GSTT2B	glutathione S-transferase T2B	0.12	ENOX1	ecto-NOX disulfide-thiol exchanger 1	−0.13
PRDX6	peroxiredoxine 6	0.11	NOX5	NADPH oxidase 5	−0.10
DUOX1	Dual oxidase 1	0.10	CAT	catalase	−0.09
GPX5	glutathione peroxidase 5	0.10	GSTA3	glutathione S-transferase A3	−0.08
PRDX1	peroxiredoxine 1	0.09	TXNDC2	thioredoxin domain-containing protein 2	−0.07
GSR	glutathion reductase	0.08	TXNDC17	thioredoxin domain-containing protein 17	−0.07
PRDX3	peroxiredoxine 3	0.05	GSTO1	glutathione S-transferase omega 1	−0.05
GSS	glutathion synthetase	0.05	GSTA2	glutathione S-transferase A2	−0.02
TXNDC11	thioredoxin domain-containing protein 11	0.04	TXNDC15	thioredoxin domain-containing protein 15	−0.01
GSTM3	glutathione S-transferase Mu 3	0.03	GSTM1	glutathione S-transferase Mu 1	−0.01
GSTA4	glutathione S-transferase A4	0.02			
GSTM4	glutathione S-transferase Mu 4	0.02			
SOD3	super oxide dismutase 3	0.01			
GPX3	glutathione peroxidase 3	0.01			
GPX1	glutathione peroxidase 1	0.00			

## 3 Discussion

H_2_O_2_ is the most investigated and best-understood oxidant in redox biology. As a result, much is known about its effects and toxicity. The cellular profiles associated *a priori* with the sensitivity to H_2_O_2_-induced cellular demise are less explored, however. Performing a 35 cell line H_2_O_2_ sensitivity screening correlated with culture-matched in-house transcriptomics enabled us to shed light on genes and pathways associated with H_2_O_2_ toxicity.

The overarching goal of this study was to provide clues on pathways and targets associated with H_2_O_2_ sensitivity. More than 500 genes correlated to this parameter with high significance, many related to cell cycle progression. This underlines a previous report showing cell cycle phases affecting H_2_O_2_-induced apoptosis. Compared to the GO/G1 phase, K562 cells were more or less resistant to H_2_O_2_ in the S-phase and G2/M phase, respectively [15]. The higher resistance to H_2_O_2_-mediated toxicity was also found in GO/G1 phase C10 cells [16]. We did not analyze cell cycle phases in detail in this study but found cells under serum-starving conditions, known to induce G0/G1 cell cycle arrest [17], to be less sensitive to oxidant-induced demise, albeit serum starvation is known to drastically change gene and protein expression and phosphorylation of processes unrelated to cell cycling [18,19]. In addition, the idea of redox control in DNA replication [20] and cycle progression [21] has long been postulated. For instance, the transcription factor FOXO1 was found to increase antioxidant defense while suppression of FOXO3a signaling abrogated H_2_O_2_-induced signaling, leading to markedly declined cell death upon pharmacological and genetic augmentation and repression, respectively [22]. Notably, activation of FOXO transcription factors directly contributes to cell cycle arrest [23], making its high or low constitutive activity a possible explanation to our findings across the 35 cell lines investigated. Moreover, oxidation states of peroxiredoxins have been linked to cell cycle progression [24]. Interestingly, genetic suppression of peroxiredoxin II increased cell cycle activity and doubling time in glioblastoma cells and simultaneously elevated H_2_O_2_ and radiation-mediated cell death [25]. Together with our data, these findings support the notion that active cell cycling sensitizes tumor cells to oxidant-induced cell death. Another intriguing hypothesis is epigenetic imprinting of low-dose H_2_O_2_ [26], opening up the idea that differing constitutive endogenous H_2_O_2_ levels might dictate the cell death amplitude towards exogenous H_2_O_2_ exposure.

Examining cancer cell sensitivity towards drugs and other effectors is a field of heavy investigation, especially at the genomic level [27]. At the same time, a myriad of molecules is involved in regulating or dysregulating cell cycle progression and control in cancer [28]. H_2_O_2_ is a ubiquitously generated and long-lived oxidant with significant contributions to many of these processes via protein thiol-mediated signaling [29] employed in several catalytic reactions involving, for instance, heme and thiol peroxidases [30]. Gene expression screenings might not fully encompass the importance of cellular signaling as this is governed by enzymatic activities of phosphorylation/de-phosphorylation and oxidation/reduction. Hence, we do not propose the main targets identified in this study to cause H_2_O_2_ sensitivity or resistance directly but rather to be a consequence of individual upstream signaling. Some of the targets, however, have been implicated in cancer and cell death related to oxidants. Tubulin (TUBB) is a druggable target [31] as it has been ascribed a role in tumorigenesis and metastasis of many cancer entities [32] and is critically regulated by Stathmin1 (STMN1) [33]. Increased Stathmin1, in turn, is associated with several types of cancer, including melanoma [34], presumably to manage microtubule formation in highly proliferating cells [35]. Stathmin 1 controls cell cycle progression via G1 to S and G2 to M checkpoint control. Increased Stathmin1 expression is associated with poor prognosis in many cancers [36,37]. Importantly, its activity is regulated via phosphorylation such as MAPK, p53, and LMP1 [38], which was not measured in our study, limiting conclusions on transcript levels alone. Nevertheless, the general finding in the literature is that increased Stathmin1 expression associates with elevated proliferation and migration. Transcriptional regulation of stathmin 1 is facilitated via Fork-head box protein M1 [39]. Elevated Denticleless (DTL) expression is associated with poor survival in different tumor types [40,41]. Albeit we could not confirm its correlation at the protein level in six selected cell lines, its gene expression changed during starvation experiments, e.g., in A549 cells. Interestingly, a recent study comparing control and H_2_O_2_-treated A549 cell transcriptomes identified DTL as the second top hit regulated by the treatment and confirmed its importance in lung cancer progression [42]. SIRPG is one of the ligands of CD47 [43], a molecule heavily investigated in clinical immuno-oncology [44]. Yet, a direct role of SIRPG tumor cell maintenance or redox process is not evident, making its appearance in our most correlating genes interesting for future studies. NCAPG has also spurred interest in being a possible anti-meiosis and mitosis target in cancer therapy [45], but little is known about, e.g., H_2_O_2_-mediated effects. CDC25A is a nuclear phosphatase regulating several CDK family members to allow cell cycle progression and found to be frequently overexpressed in cancers [46]. It is subject to redox regulation [47]. Elevated MCM10 levels are associated with poor prognosis in cancer patients [48,49], but a redox-related upstream control has not been described [50]. The higher-than-average expression of these cell cycle-related genes across many H_2_O_2_ cell lines used in our study remains remarkable, nevertheless. The oxidoreductase biliverdin (BLVRB) is a redox-regulated enzyme involved in heme catabolism and Fe^3+^ reduction [51]. BLVRB is a transcriptional target of Nrf2 and showed the highest positive correlation among all genes in our study, potentially protecting H_2_O_2_-resistant cell lines by increasing levels of the free radical scavenger bilirubin [52].

Albeit being overall heterogeneously expressed, several transcription factors (TF) showed a good (Spearman r > 0.50 or < -0.50) correlation with H_2_O_2_-mediated toxicity. Three members of the E2F family emerged as top negatively correlating TF. Reduced E2F1 levels or its inactivation leads to reduced cell cycling and inhibition of G1/S transition [53,54]. Thus, its negative correlation is in line with the negative correlation of the other cell cycle related genes identified in our study. Along similar lines, the overall reduced expression of E2F2 in H_2_O_2_-resistant cell lines in our study would be expected to be associated with decreased cell cycling [55]. E2F7 can both antagonize E2F1-induced proliferation [56] as well as promote cell-cycle progression and proliferation [57]. Enhanced E2F7 expression is also found in many types of cancer and a predictor of poor prognosis, e.g., in OSCC [58] and glioblastoma patients [59], and for E2F1/2/7/8 also in cervical cancer patients [60]. Regarding the TF with good positive correlation to H_2_O_2_-mediated toxicity, two KLF (Krüppel-like factor) family members (KLF4 and KLF5) constituted the top genes. Both TFs regulate proliferation, and it was shown that KLF4 and KLF5 overexpression sensitized cells to H_2_O_2_-induced toxicity [61]. This is line with our study where, in tendency, H_2_O_2_-sensitive cell lines expressed higher KLF4 and KLF5 levels than resistant cell lines. KLF4 is frequently decreased or lost in epithelial cancers [62], attributing it a role as tumor suppressor [63]. KLF5, in turn, is though to promote tumorogenesis by activating cell cycle-related genes [64] and acting as mediator of responses to external stressors [65]. However, also cell differentiation-dependent effects of KLF5 are described accelerating proliferation in non-transformed cells and vice versa in transformed cells [66]. NFIL3 (nuclear factor interleukin 3-regulated, also known as E4BP4) correlated negatively in our study. NFIL3 is increased in many types of cancer [67] by restricting FOXO chromatin access, and is associated with enhanced tumor cell survival [68] and resistance to H_2_O_2_-mediated cell death [69].

Among the ROS-related and antioxidant genes, GPX2 and NOXA1 and GPX7 and NOS had a good (Spearman r > 0.40 or < -0.40) positive and negative correlation, respectively, with H_2_O_2_-mediated toxicity (Table 4). GPX2 is cytoprotective by reducing H_2_O_2_ and hence expected to be increasingly expressed in resistant cell lines in our study. It is frequently found to be increased in tumors [70,71]. GPX7 is found in the lumen of the endoplasmic reticulum and besides reacting with H_2_O_2_, it interacts with PDI family members to support protein folding [72], and serves as stress sensor [73]. Hence, it is conceivable that it functions in supporting growth in the H_2_O_2_-sensitive cells in our study with enhanced cell cycle gene-signatures to maintain homeostasis and proteostasis. The positively correlating target NOXA1 is a co-factor of NOX1 (especially in colon cells) [74], suggesting increased NOX1 activity in H_2_O_2_-resistant cells in our study. Inducible NOS (NOS2) negatively correlated in our study and is heavily involved in tumor progression and metastasis and different cancer types due to its regulation by p53 [75]. The reasons of why increased nitric oxide production is associated with increased sensitivity to H_2_O_2_-mediated cellular demise is unclear. Notwithstanding, its noteworthy mentioning the value of target genes associated with increased sensitivity to stressors as this provides straightforward testable hypotheses to gain a better mechanistic understanding.

Our study had several limitations. First, cells’ states and regulation is governed by many processes, such as thiol oxidation, phosphorylation, epigenetic changes, micro RNAs, oxidative posttranslational modifications, and protein expression that were not covered in our work. Nevertheless, our transcriptomics-based approach identified provides testable hypotheses and appropriate cell lines to elucidate the exact roles of cell cycle control and proliferation and H_2_O_2_-induced toxicity further. Second, conclusions from our serum-starving approach are limited due to the massive cellular changes that come with starvation. Instead, a thymidine block might be more suitable combined with in depth cell cycle analysis. Yet, it should be kept in mind that different agents, stressors, or inhibitors designed to change the basal expression of genes and proteins may not only have off-target effects but might also be differently effective in different cell types. The approach in our study is simplistic but therefore remains void of additional perturbations. Third, interconnected to the previous points, we did not measure responses to H_2_O_2_ but only gene expression of untreated cells. It would be interesting to study such response but many uncertainties are connected with such an approach, e.g., timing of analysis (minutes to hours) interconnected with level of analysis (gene, post-transcriptional, protein, post-translational, epigenetic, etc.), which is difficult to do across larger panels of cell lines. A fourth limitation is the lack of one or several clear-cut candidates potentially explaining the effects, as even the highly correlating genes had some level of heterogenous expression, and the missing functional verification of the targets. However, keeping the different levels of cellular regulation in mind, we did not aim to proof the highly correlating genes to be causative for the effects observed. Instead, we propose an association of cell cycle signaling pathways being important for cellular survival to H_2_O_2_, while the exact genes and proteins crucial to this process might differ for each cell type as seen in the expression heatmaps.

## 4 Conclusion

This study aimed at identifying in-house generated gene expression signatures *a priori* associated with H_2_O_2_-mediated toxicity in 34 tumor cell lines of different entities. Apart from several pathways identified, many highly correlating genes were significantly related to the cell cycle in demarcating H_2_O_2_ sensitivity. As the exact mechanism remains unclear, further understanding of the intertwined relationships between redox signaling and regulated cell death during active or suppressed cell cycling may aid in generating an overarching scheme of how to effectively redox-targeted tumor cells.

## 5 Materials and methods

### 5.1 Cell culture

Thirty-five cell lines of different origins were used in this study. This included melanoma (SK-MEL-19, SK-MEL-29, SK-MEL-63, SK-MEL-147, MaMel-86a, MeWo, 501-Mel, and A375), leukemia (THP-1, Jurkat, TK6, U937, and HL-60), adenocarcinoma (A431, Capan-1, HeLa, HT-29, MCF7, MDA-MB-231, OVCAR-3, A549, MIA-PaCa-2, PaTu-T, PaTu-S, PC3, SK-OV-3, SW480, TOV-112D, and TOV-21G) and squamous cell carcinoma (SCC-13, SCL-1, and SCL-2) cell lines. In addition, the neuroblastoma cell lines SH-SY5Y and the non-malignant keratinocyte cell line HaCaT were used. HeLa and its derivative HeLa 2c were kindly provided by Christopher Lillig (Greifswald University Medical Center, Germany). All cell lines were maintained in their respective culture media, maintained under standard cell culture conditions, and passaged 2–3x per week.

### 5.2 Metabolic activity

To determine the metabolic activity, 1 × 10^4^ cells were seeded in 100 μl of fully supplemented cell culture medium (RPMI1640 containing 10% fetal bovine serum, 1% glutamine, and 1% penicillin/streptomycin) per well in 96-well plates (Eppendorf, Germany). The plates were equipped with a rim filled with deionized water to provide extra evaporation protection and keep the edge effect at a minimum. Eighteen hours later, the cells received different concentrations of H_2_O_2_ (1 μM, 10 μM, 100 μM, or 1000 μM). After 20 h of incubation, resazurin (final concentration: 100 μM) was added to each well, and the cells were cultured for another 4 h. Subsequently, resorufin fluorescence was recorded using a bandpass filter-based multimode plate reader (Tecan F200) at *λ*_ex_ 560 nm and *λ*_em_ 590 nm. After background subtraction, data normalization was performed against the untreated controls. The experimental setup for metabolic activity determination was the same for starvation experiments, except that previously expanded fetal bovine serum-starved cells were used. H_2_O_2_ concentrations were log-transformed and displayed against normalized metabolic activity rates. IC_25_ values were calculated using non-linear least squares fit based on asymmetric confidence intervals. The IC_25_ was chosen over the IC_50_ for two interconnected reasons. First, we were interested in less drastic concentrations of H_2_O_2_ as they appear physiologically or realistically within therapeutic regimens. The maximum concentration of 1000 μM employed in our screening is already beyond this. Second, 34 out of 35 cell lines reached the IC_25_ based on our measurements, while this was not the case for nearly half the cell lines when testing for the IC_50_. In the latter case, extensive interpolation of IC_50_ values would have been needed, potentially introducing additional uncertainty regarding correlation analysis that was to aim to avoid.

### 5.3 Transcriptomics

For transcriptomic analysis, at least 5 × 10^5^ cells were grown in culture flasks and harvested using accutase. Cells were washed, and total RNA was isolated using the Mini RNA purification kit (Bio&Sell). RNA extracts were incubated with RNase-free DNase Ι to remove contaminating DNA. RNA concentrations were then quantified via spectrophotometry (Nanodrop 2000) and stored at −80 °C. Sample preparation and hybridization were performed according to a single-color, chip-based transcriptome microarray gene expression analysis protocol. First, the spike-in mix was added to 100 ng of RNA, and the RNA was transcribed into complementary DNA (cDNA). cDNA was amplified using the Low Input Quick Amp Labeling One Color component. Next, the cDNA was transcribed into complementary RNA while incorporating the fluorescent dye cyanine 3 (Cy3). After purification, the Cy3-cRNA was fragmented, and hybridization onto a microarray chip was performed using the Gene Expression Hybridization Kit (SurePrint G3 Human CGH Microarray 8x60; Agilent) for 17 h at 65 °C. The chips were washed using the Gene Expression Wash Buffer Kit (all from Agilent), dried in a dedicated microarray chip oven, and scanned using an Agilent SureScan device. The scan data were extracted using Agilent's Feature Extraction Software and analyzed by the GeneSpring software (Agilent Technologies, Santa Clara, CA, USA). Three microarray samples were sampled from three independent biological replicates for each cell line, totaling 105 transcriptomes averaged to 35.

### 5.4 Bioinformatics

Transcriptomic microarray raw data files were loaded into GeneSpring and RMA-normalized before normalizing expression against RPL13A as housekeeping gene [76]. Hierarchical clustering of gene expression across all cell lines was performed using Perseus 1.65 software. Relative gene expression was then Spearman correlated against the predetermined H_2_O_2_ IC_25_ values using TipCo Spotfire 7.8 software (PerkinElmer). Only genes with a Spearman r > 0.50 or r < −0.50 are shown; these genes always correlated significantly. Significantly correlating genes with Spearman r values different from these ranges were not included. Principal component analysis was realized using TipCo Spotfire software as well. Functional gene analysis was done using Gene Ontology (GO) pathway analysis and the *Protein Analysis Through Evolutionary Relationships* (PANTHER) classification system. Additionally, pathway analysis was performed using ShinyGo (an online open-access software tool). STRING database was used to identify co-expressed targets and shared networks. In this work, ‘target’ refers to genes identified from our analysis and not the protein being directly oxizided by H_2_O_2_.

### 5.5 Flow cytometry

Cells were fixed in ice-cold methanol, washed, and stained with 4′,6-diamidino-2-phenylindole (DAPI, 10 μM; BioLegend) for 1 h. After washing, cells were resuspended in PBS with DAPI and measured using flow cytometry (CytoFLEX S; Beckman-Coulter). Analysis was performed using Kaluza software 2.1 (Beckman-Coulter) using the Michael H. Fox algorithm to identify cells in the G1 phase of the cell cycle.

### 5.6 qPCR

Total RNA (1 μg) in a total volume of 20 μl of buffer was reverse-transcribed, and qPCR was done using a QuantStudio 1 device (Thermo Fisher Scientific). GAPDH (Glyceraldehyde 3-phosphate dehydrogenase) was used as a housekeeping gene and normalization control. Cycling parameters were: 95 °C for 1 min to activate the DNA polymerase, then 40 cycles of denaturation for 10 s at 95 °C, annealing for 15 s at 60 °C, and extension for 20 s at 72 °C. Single product formation was confirmed by melting point analysis. Samples were run in duplicate, and the _ΔΔ_CT method was applied to calculate relative expression intensities where treated and untreated samples were compared. For assessment of baseline gene expression, reciprocal _Δ_CT values were calculated.

### 5.7 Western blot

Cells were pelleted and lysed in RIPA buffer containing protease and phosphatase inhibitors (cOmplete Mini, phosSTOP, PMSF; Sigma-Aldrich). Protein expression levels of β-tubulin, SIRP-γ, DTL, NCAPG, MCM10, CDC25A, and ME1 were determined using appropriate monoclonal antibodies (Cell signaling) and capillary-based gel electrophoresis performed by the *WES* system (ProteinSimple) according to the manufacturer's instructions. Band intensities were quantified using *Compass for Simple Western* Software and normalized to housekeeping control protein expression (GAPDH) for six cell lines (Jurkat, TK6, PaTu-T, PaTu-S, A549, and Capan-1) investigated.

### 5.8 Statistical analysis

The experiments were repeated three independent times. Statistical analysis was performed using prism software 9.2. Analysis for gene and protein expression in relation to IC_25_ was done using nonparametric, two-tailed Spearman correlation with 95% confidence intervals. Comparison between two groups for qPCR results was made using unpaired, two-tailed *t*-test. Statistically significant results were indicated as p < 0.05 (*), p < 0.01 (**), and p < 0.001 (***).

## Funding

Funding was received by the 10.13039/501100002347German Federal Ministry of Education and Research (BMBF), grant numbers 03Z22DN11, 03Z22DN12, and 03Z22Di1.

## Declaration of competing interest

The authors declare no conflict of interest.

## Appendix A Supplementary data

**Supplementary Table 1: Correlation of transcription factor gene expression to H**_**2**_**O**_**2**_**IC**_**25**_**values.** Given are genes and protein names of transcription factors positively (left) and negatively (right) correlating to H_2_O_2_ IC_25_ values. Grey shading: Spearman r correlation >0.5 and <-0.5.

**Supplementary Fig. 1: PCA.** Principal component analysis of transcriptomic profiles of 35 cell lines included in the study.

**Supplementary Fig. 2: Positively correlating gene expression per cell line.** Heat map of positively (r > 0.50) and significantly (p > 0.01) correlating genes sorted descending in relation to H_2_O_2_ IC_25_ (from left to right).

**Supplementary Fig. 3: Negatively correlating gene expression per cell line.** Heat map of negatively (r < −0.50) and significantly (p > 0.01) correlating genes sorted descending in relation to H_2_O_2_ IC_25_ (from left to right).

**Supplementary Fig. 4: STRING network analysis.** Co-expression maps of top 10 targets identified and expression network of six targets intertwined in cell cycle pathways.

**Supplementary Fig. 5: Functional verification.** (**a**) ratio of metabolic activity from untreated starved over untreated serum-cultured cell lines; (**b**) representative flow cytometry histograms of serum-cultured and starved cells at 24 h using DAPI to stain DNA for algorithm-driven cell cycle analysis; (**c**) log-transformed H_2_O_2_ concentrations plotted against normalized metabolic activity rates of serum-cultured or starved cells 24 h after exposure to H_2_O_2_, and non-linear regression analysis to identify the IC_25_ for each cell line with the fold-change of serum-cultured over starved conditions given within the graphs in black; (**d**) gene expression (qPCR) of two experiments given as the ratio of starved over serum-cultured cell lines. Data (a,c) are mean of three experiments.

**Supplementary Fig. 6: Gene expression of ROS-related proteins.** Relative expression normalized to the mean expression of each gene across all 35 cell lines sorted for positive and negative correlation (r) against the H_2_O_2_ IC_25_ of each cell line.
