# Supplementary material for: Cell cycle-related genes associate with sensitivity to hydrogen peroxide-induced toxicity
Source: Redox Biol. 2022 Jan 17;50:102234. doi: 10.1016/j.redox.2022.102234 (PMC8783094; doi:10.1016/j.redox.2022.102234)

**Cell cycle-related genes associate with sensitivity to hydrogen peroxide-induced toxicity**

*Sander Bekeschus^1^, Grit Liebelt^1^, Jonas Menz^1,2^, Debora Singer^1^, Kristian Wende^1^, Anke Schmidt^1^

1 ZIK *plasmatis*, Leibniz Institute for Plasma Science and Technology (INP), Felix-Hausdorff-Str. 2, 17489 Greifswald, Germany

2 Department of General, Visceral, Vascular, and Thorax Surgery, Greifswald University Medical Center, Felix-Hausdorff-Str. 2, 17475 Greifswald, Germany

* correspondence: sander.bekeschus@inp-greifswald.de

Keywords: cancer; hydrogen peroxide; oxidative stress; reactive oxygen species; ROS

Supplemental Tables and Figures.

## Table S1

| **Gene** | **Protein** | **r (+)** | **Gene** | **Protein** | **r (-)** |
| --- | --- | --- | --- | --- | --- |
| KLF5 | kruppel like factor 5 | 0.55 | E2F1 | transcription factor activator | -0.58 |
| KLF4 | kruppel like factor 4 | 0.51 | E2F2 | transcription factor activator | -0.57 |
| NFIL3 | nuclear factor, interleukin 3 regulated | 0.50 | E2F7 | transcription factor suppressor | -0.50 |
| ELF3 | E74 Like ETS transcription factor 3 | 0.48 | KLF17 | kruppel like factor 17 | -0.47 |
| KLF3 | kruppel like factor 3 | 0.41 | TRIM28 | transcription intermediary factor 1-beta | -0.45 |
| NFE2L1 | nuclear factor erythroid 2-related factor1 (NRF1) | 0.36 | TFAP2E | activating enhancer binding Protein 2 epsilon | -0.42 |
| KLF10 | kruppel like factor 10 | 0.34 | NFYA | nuclear transcription factor Y subunit alpha | -0.42 |
| JUN | Protein: AP-1 | 0.32 | E2F8 | transcription factor suppressor | -0.41 |
| NFIB | nuclear factor 1 B-type | 0.30 | TFAP2D | activating enhancer binding Protein 2 delta | -0.41 |
| TFAP2A | activating enhancer-binding Protein 2 alpha | 0.29 | NFATC3 | nuclear factor of activated T-cells, cytoplasmic 3 NFAT4! | -0.40 |
| KLF8 | kruppel like factor 8 | 0.29 | NFATC2 | nuclear factor of activated T-cells, cytoplasmic 2 NFAT1! | -0.39 |
| TEAD3 | TEA domain transcription factor 3 | 0.28 | TFEC | transcription factor EB | -0.37 |
| KLF11 | kruppel like factor 11 | 0.28 | TFDP1 | transcription factor Dp-1, E2F family | -0.36 |
| NFX1 | transcriptional repressor NF-X1 | 0.25 | SP1 | SP1 transcription factor | -0.32 |
| TFCP2L1 | transcription factor CP2 Like 1 | 0.23 | E2F3 | transcription factor activator (3a), suppressor (3b) | -0.32 |
| NFKBIZ | NFKB inhibitor zeta | 0.23 | TFDP3 | transcription factor Dp-3, E2F family | -0.32 |
| NFKBIA | NFKB inhibitor alpha | 0.20 | KLF15 | kruppel like factor 15 | -0.29 |
| TFAP4 | activating enhancer-binding protein 4 | 0.20 | NFYB | nuclear transcription factor Y subunit beta | -0.27 |
| NFXL1 | nuclear transcription factor, X-box binding ligand 1 | 0.18 | NFE4 | nuclear factor, erythroid 4 | -0.27 |
| KLF7 | kruppel like factor 7 | 0.18 | TFAP2B | activating enhancer-binding Protein 2 beta | -0.26 |
| TFAP2C | activating enhancer binding Protein 2 gamma | 0.16 | E2F6 | transcription factor suppressor | -0.26 |
| TFB2M | dimethyladenosine transferase 2; transcription factor B2 | 0.15 | KLF12 | kruppel like factor 12 | -0.24 |
| ELF4 | E74 Like ETS transcription factor 4 | 0.13 | KLF1 | kruppel like factor 1 | -0.21 |
| TFE3 | transcription factor E3 | 0.12 | NFIC | nuclear factor 1 C-type | -0.20 |
| NFE2 | transcription factor NF-E2 45 kDa subunit | 0.10 | TP73 | tumor suppressor protein 73 | -0.20 |
| NFIA | nuclear factor 1 A-type | 0.09 | NFKBID | NFKB inhibitor delta | -0.19 |
| TFCP2 | alpha-globin transcription factor CP2 | 0.06 | ELF2 | E74 Like ETS transcription factor 2 | -0.17 |
| TEAD1 | TEA domain transcription factor 1 | 0.05 | NFATC4 | nuclear factor of activated T-cells, cytoplasmic 4 NFAT3! | -0.17 |
| KLF9 | kruppel like factor 9 | 0.05 | NFYC | nuclear transcription factor Y subunit gamma | -0.16 |
| NFAT5 | nuclear factor of activated T-cells, cytoplasmic 4 | 0.04 | NFKBIE | NFKB inhibitor epsylon | -0.16 |
| E2F5 | transcription factor suppressor | 0.03 | TEAD4 | TEA domain transcription factor 4 | -0.15 |
| KLF13 | kruppel like factor 13 | 0.01 | TEAD2 | TEA domain transcription factor 2 | -0.14 |
| KLF6 | kruppel like factor 6 | 0.00 | ELF1 | E74 Like ETS transcription factor 1 | -0.13 |
|  |  |  | NFKBIB | NFKB inhibitor beta | -0.12 |
|  |  |  | KLF14 | kruppel like factor 14 | -0.12 |
|  |  |  | TP53 | tumor suppressor protein 53 | -0.12 |
|  |  |  | TFEB | transcription factor EC | -0.10 |
|  |  |  | E2F4 | transcription factor suppressor | -0.09 |
|  |  |  | NFRKB | nuclear factor related to kappa-B-binding protein | -0.08 |
|  |  |  | NFE2L3 | nuclear factor erythroid 2-related factor 3 (NRF3) | -0.07 |
|  |  |  | NFIX | nuclear factor 1 X-type | -0.06 |
|  |  |  | KLF16 | kruppel like factor 16 | -0.06 |
|  |  |  | NFKB2 | nuclear factor kappa-light-chain-enhancer of activated B cells | -0.05 |
|  |  |  | TP63 | tumor suppressor protein 63 | -0.04 |
|  |  |  | NFE2L2 | nuclear factor erythroid 2-related factor 2 (NRF2) | -0.03 |
|  |  |  | NFKB1 | nuclear factor kappa-light-chain-enhancer of activated B cells | -0.03 |
|  |  |  | TFDP2 | transcription factor Dp-2, E2F family | -0.03 |
|  |  |  | KLF2 | kruppel like factor 2 | -0.02 |
|  |  |  | ELF5 | E74 Like ETS transcription factor 5 | -0.01 |
|  |  |  | NF1 | nuclear factor, erythroid 1 | -0.01 |
|  |  |  | NFATC1 | nuclear factor of activated T-cells, cytoplasmic 1 NFAT2! | -0.01 |
|  |  |  | TFAM | mitochondrial transcription factor A | -0.01 |

## Figure S1


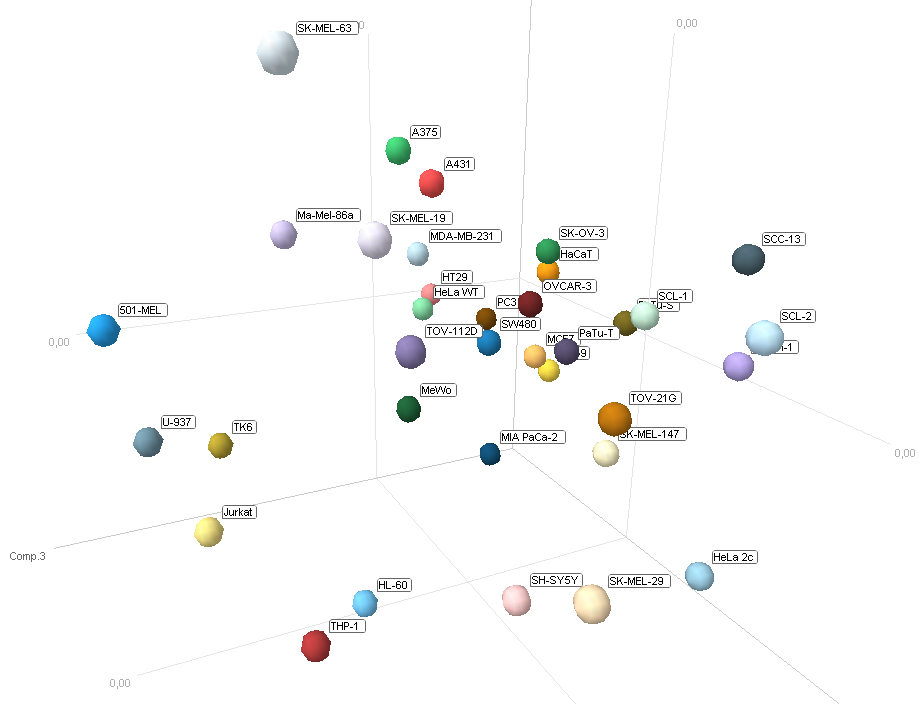


## Figure S2

## Figure S2 (continued)

## Figure S3

## Figure S3 (continued)

## Figure S3 (continued)

## Figure S3 (continued)

## Figure S4


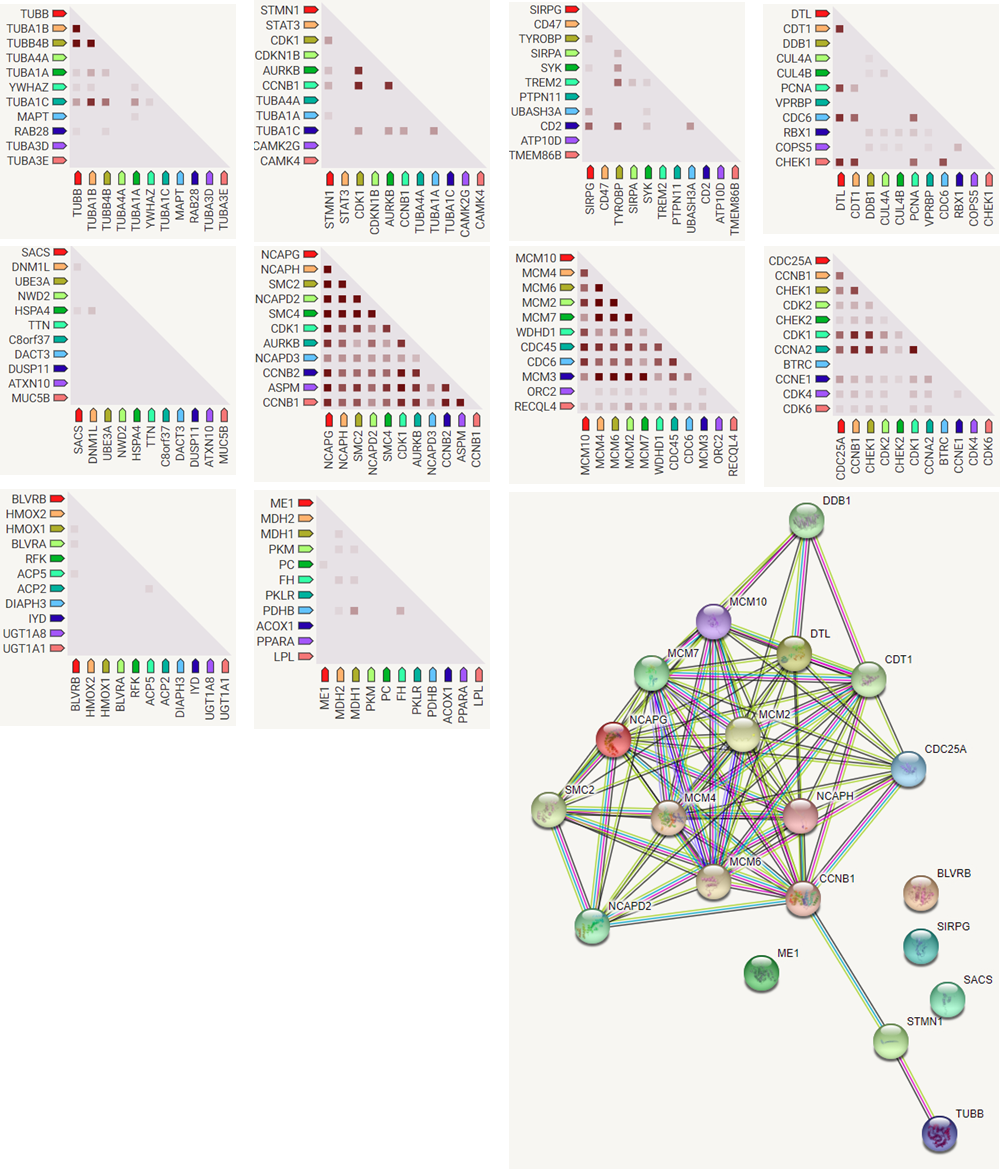


## Figure S5


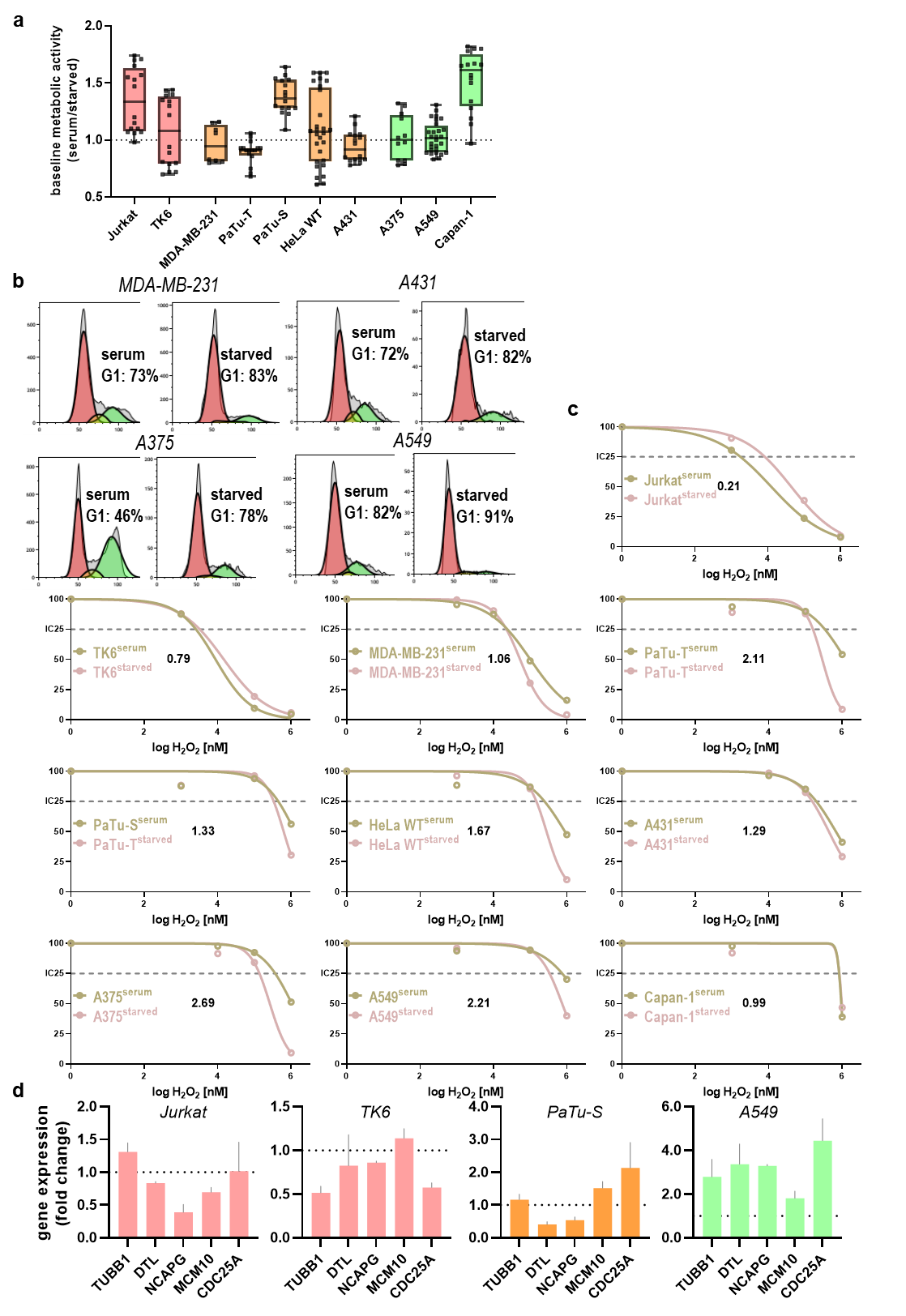


## Figure S6


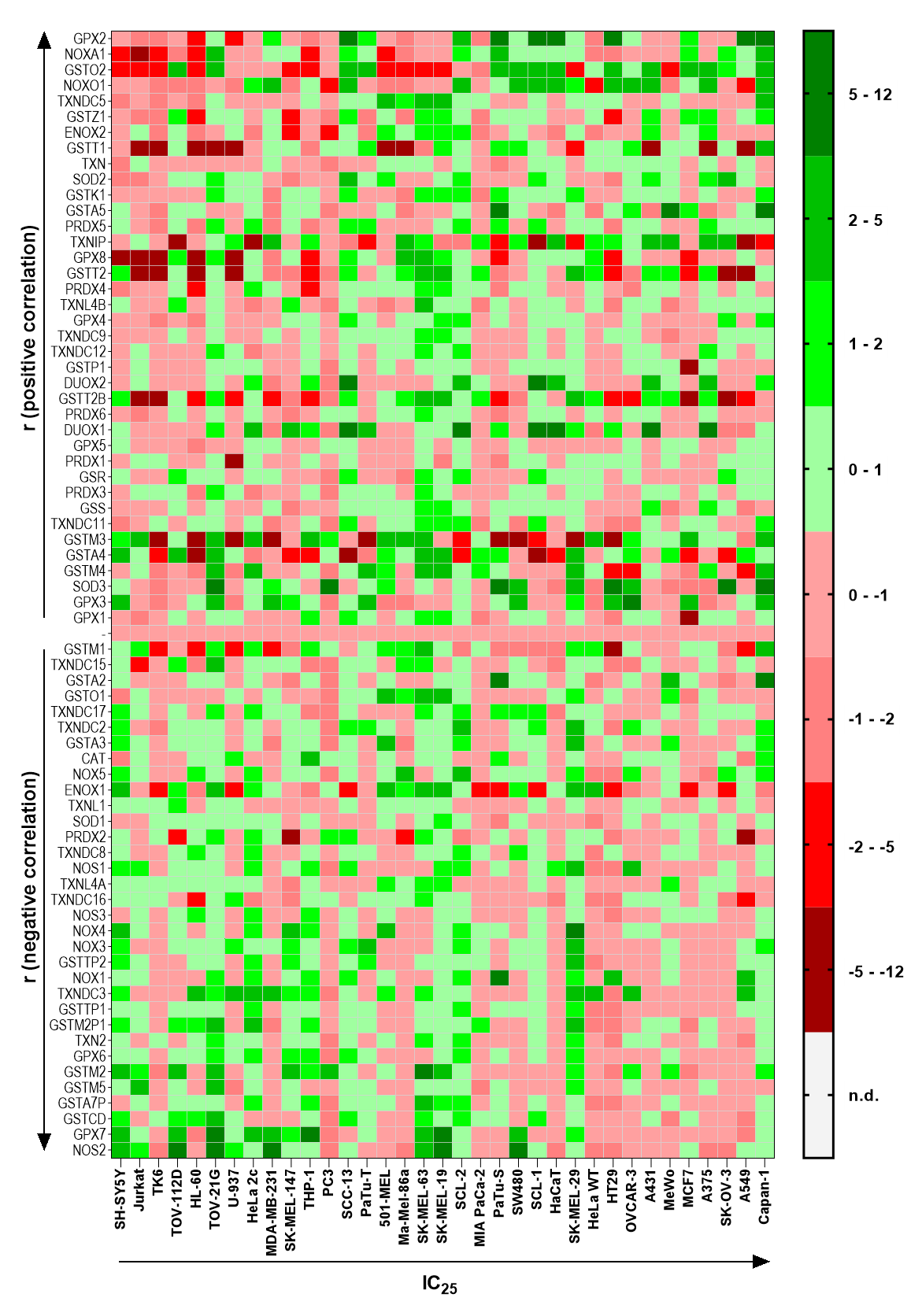

Supplement: Multimedia component 1 [file mmc1.docx]
